# A study on fluoroscopic images in exposure reduction techniques ― Focusing on the image quality of fluoroscopic images and exposure images

**DOI:** 10.1002/acm2.12549

**Published:** 2019-04-01

**Authors:** Hisaya Sato, Daisuke Kittaka, Miwa Ohsawa, Kyoichi Kato

**Affiliations:** ^1^ Showa University Graduate School of Health Sciences Tokyo Japan; ^2^ Department of Radiological Technology Showa University Hospital Tokyo Japan; ^3^ Department of Radiological Technology Showa University Fujigaoka Hospital Yokohama Japan; ^4^ Department of Unification Radiological Technology Showa University Tokyo Japan

**Keywords:** exposure image, fluoroscopic image, gray value, image quality

## Abstract

The quality of the present day fluoroscopic images is sufficiently high for use as exposure images depending on the environment where the fluoroscopic images are recorded. In some facilities which use fluoroscopic images as exposure images they are recorded with a radiological x‐ray diagnostic device equipped with a fluoroscopic storage function. There are, however, cases where fluoroscopic images cannot be used as exposure images because the quality of the fluoroscopic image cannot be assured in the environment where the fluoroscopic images are recorded. This poses problems when stored fluoroscopic images are used in place of exposure images without any clearly established standard. In the present study, we establish that stored fluoroscopic images can be used as exposure images by using gray values obtained from profile curves. This study finds that replacement of stored fluoroscopic images with exposure images requires 20.1 or higher gray scale value differences between the background and signal, using a 20 cm thick acrylic phantom (here an adult abdomen as representing the human body) as the specific geometry. This suggests the conclusion that the gray value can be considered a useful index when using stored fluoroscopic images as exposure images.

## 1 INTRODUCTION

One feature in the treatment of cardiac catheterization is the repeated fluoroscopy and imaging required during treatment. Fluoroscopic time (exposure), particularly when dealing with complicated diseases is prolonged,^1^, ^2^, ^3^ and the number of times where imaging is required tends to increase. This increases the dose which patients are exposed to during the treatment, resulting in a higher risk of skin injury due to radiation.^4^, ^5^

The reason for the lengthening of the fluoroscopy time is that it takes time to orient the guidewire to the intended direction when inserting the guidewire used for intravascular treatment.^6^, ^7^ Fluoroscopic observations take time because the physician is observing the distal end of the guidewire throughout the treatment. The need for increases in the number of occasions needing fluoroscopy arises as, in the course of the treatment, physicians advance the guidewire millimeter by millimeter, and record images whenever the position of the guidewire changes. Imaging serves the purpose to record the progress of the treatment being the record used for treatment assessments of the blood vessel in the diseased area and as image data for repeated observations following the treatment. For this reason, the imaging dose is set higher than the fluoroscopy dose.^8^

Images are also used in documenting that appropriate treatment has been performed. However, the quality of the present day fluoroscopic images is sufficiently high for use as exposure images depending on the environment where the fluoroscopic images are recorded,^9^ and some facilities that use fluoroscopic images as exposure images employ a radiological x‐ray diagnostic device equipped with a fluoroscopic storage function. In addition, there are cases where fluoroscopic images cannot be used as exposure images because the quality of the fluoroscopic image cannot be ensured in the environment where the fluoroscopic images are recorded. In this way, it poses problems when stored fluoroscopic images are used in place of exposure images without any clear established standard. In the present study, we examined whether stored fluoroscopic images can be used as exposure images by using gray scale values obtained from profile curves.

## 2 MATERIALS AND METHODS

### 2.A Definition of gray value

Digital images are collected as numerical data that need to be converted to brightness values to be observed as images. The brightness is expressed as the gray level. With x‐ray images that have 8 bits per pixel of information, values from 0 to 256 in gray scale are represented by gradations from dark (black) to light (white). The representation with numerical values using this gradation is termed the gray value.^10^, ^11^

This study uses the gray value as a value to determine balloon expansion in coronary artery treatment, as depicted in fluoroscopic and exposure images obtained with a cardiovascular x‐ray diagnostic device.

### 2.B Equipment used

For the cardiovascular x‐ray diagnosis device, we used the Allura10/10 manufactured by Philips, Innova 2100IQ manufactured by GE, and the Trinias B8 MiX package manufactured by Shimazu Co., Ltd., Hamamatsu, Japan. For the video view, we used the kada‐View manufactured by Photron M&E Solutions Inc., Tokyo, Japan, and ImageJ was used to analyze the images. We used a 20 cm thick acrylic phantom as the subject, and the area dose meter installed in the manufacturers’ equipment as the dosimeter. For visual and physical evaluations, we used the balloon for coronary artery treatment commonly used in clinical settings: 2.5/15 mm, 3.0/10 mm, 3.5/10 mm, and 4.0/10 mm (balloon diameter/balloon length) manufactured by Kaneka Corporation, and Iovelin 350 (Teva Takeda Pharma Ltd., Nagoya, Japan) as the contrast agent.

### 2.C Dose settings of x‐ray diagnostic devices for circulatory organs

**Figure 1:**
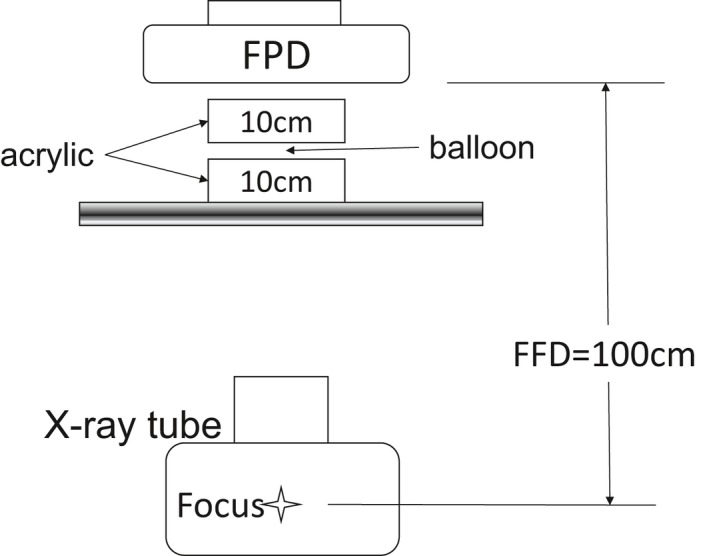
Measurement geometry of dose rate per unit time.

Using each of the tested devices, we measured the x‐ray doses of fluoroscopic and exposure images at the irradiation reference point of patients. Figure 1 shows the measurement geometry and Table 1 shows the conditions of the exposure and fluoroscopic imaging in the direction of imaging angles, Anterior‐Posterior (AP).

**Table 1:** Exposure and fluoroscopic imaging conditions by cardiovascular x‐ray diagnostic device. Imaging angle: Anterior‐Posterior (AP) direction

Device	Condition	Tube voltage (kv)	Tube current (mA)	Time (msec)
A	Exposure Fluoroscopy	81.0	200.0	5.0
		80.0	4.3	
B	Exposure Fluoroscopy	73.0	360.0	5.0
		78.0	18.7	
C	Exposure Fluoroscopy	69.0	480.0	5.0
		74.0	15.0	

### 2.D Visual evaluation of balloons at different imaging angles

The concentration of 350 mgI/mL contrast agent enclosed in a balloon for clinical coronary arteries, 4.0, 3.5, 3.0, 2.5, and 2.0 mm in diameter, was diluted twofold, and the balloon was dilated to the nominal size. The geometry of Fig. 1 was used for the exposure and fluoroscopic imaging. The imaging angles chosen were the AP direction, Right Anterior Oblique/Caudal (RAO/CAU) direction, and Right Anterior Oblique/Cranial (RAO/CRA) direction. In the visual evaluation, we determined whether the stored fluoroscopic images could be used as exposure images based on the balloon images. The evaluation was performed by eight physicians all with experience of conducting more than 300 coronary artery treatments. In the visual evaluation of the exposure and stored fluoroscopic images, we evaluated the images with the balloon dilated to the nominal size as “Suitable”, and the others as “Not suitable”.

### 2.E Physical evaluation of balloons at different imaging angles

**Figure 2:**
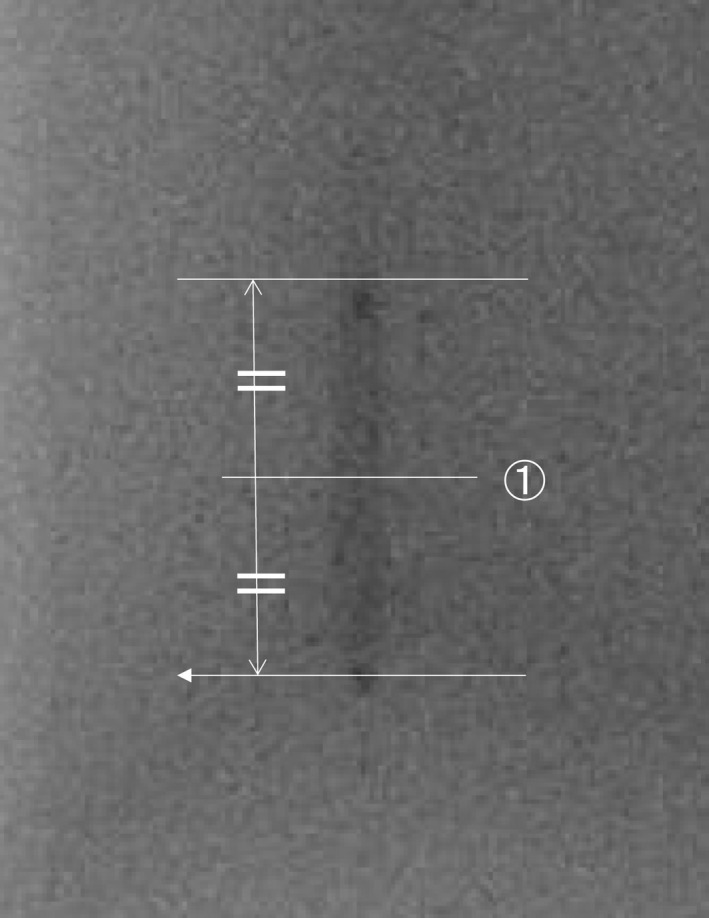
Reference point in balloon for obtaining Profile curve.

**Figure 3:**
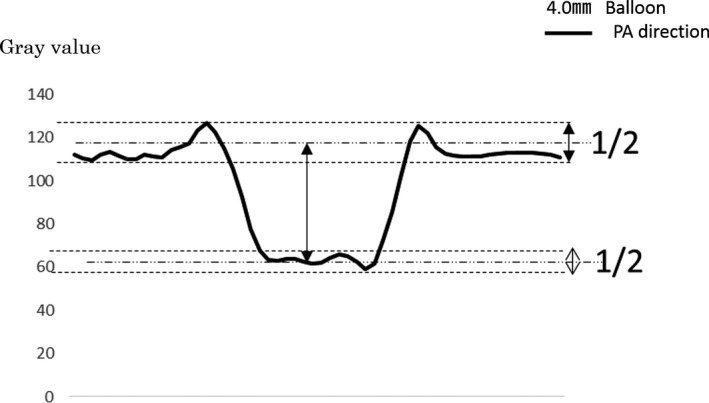
Profile curve in the PA direction of the imaging angle to obtain 4.0 mm images.

Based on the images used for visual evaluation of balloons at different imaging angles, we calculated profile curves using ImageJ. To unify the measurement points, we calculated the profile curves at position (a), the midpoint of the balloon size in the length direction as shown in Fig 2. We performed measurements five times with the profile curve as a physical evaluation, and calculated the mean value. In the physical evaluation with the profile curve, differences in the values of the background and the signal were made by the method shown in Fig. 3. In consideration of inter‐rater measurement errors, measurements were made by one medical radiologist with 26 yr of experience and certified by the Japan Professional Accreditation Board of Radiological Technologist for Angiography and Intervention.

## 3 RESULTS

### 3.A Dose settings of x‐ray diagnostic devices for circulatory organs

Table 2 shows the dose rates in the exposure and fluoroscopic imaging conditions for the cardiovascular x‐ray diagnostic devices. Considering the dose set for a fluoroscopic imaging device as the standard, the fluoroscopic doses of devices A, B, and C were 8%, 10%, and 15%, respectively.

**Table 2:** Dose rate per unit time under the exposure and fluoroscopic imaging conditions by the cardiovascular x‐ray diagnostic devices. Imaging angle: Anterior‐Posterior (AP) direction

Device	Condition	Dose rate (mGy/min)
A	Exposure Fluoroscopy	79.2
		6.7
B	Exposure Fluoroscopy	105.7
		11.0
C	Exposure Fluoroscopy	97.7
		14.8

### 3.B Visual evaluation of balloons at different imaging angles

**Figure 4:**
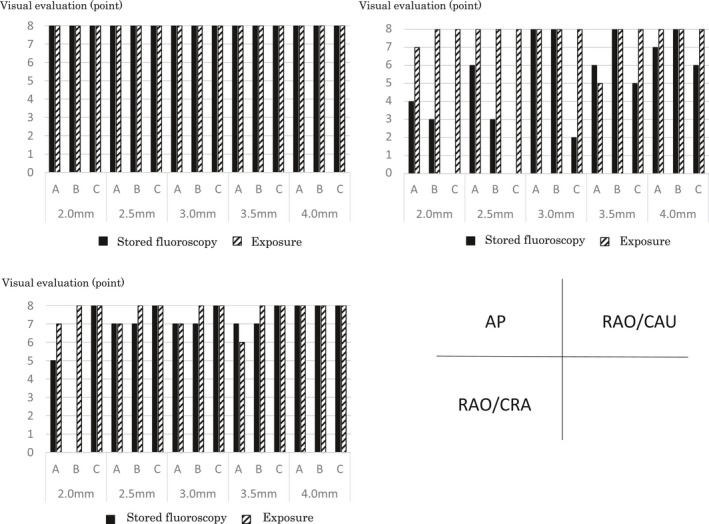
Visual evaluation of the balloon at different imaging angles by the cardiovascular x‐ray diagnostic devices.

Figure 4 shows the results of the visual evaluation of fluoroscopic and exposure images of balloons of different sizes at different angles, in the AP, RAO/CAU, and RAO/CRA directions. In the AP direction, the qualities of the stored fluoroscopic images of all balloon sizes with all devices were evaluated as similar to those of the exposure images. In the RAO/CAU direction with device C, none of the physicians were able to evaluate the stored fluoroscopic images of balloon sizes (≤2.5 mm) compared to exposure images. Also, 50% of the physicians were not able to evaluate the stored fluoroscopic images of balloon sizes (≤2.5 mm) with device B, and for the balloon size (2.0 mm) with device A. In the RAO/CRA direction, physicians were unable to evaluate the images of balloon sizes (≤2.5 mm) with device B only.

### 3.C Physical evaluation of balloons at different imaging angles

**Figure 5:**
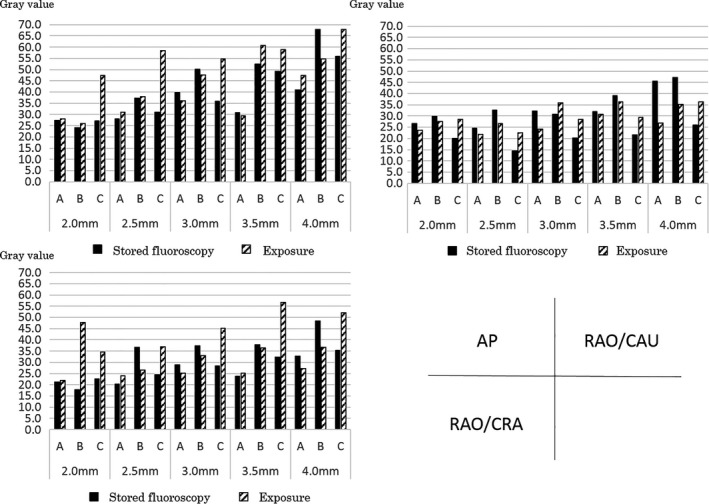
Physical evaluation of the balloon at different imaging angles by the cardiovascular x‐ray diagnostic devices.

Figure 5 shows the results of the physical evaluation of fluoroscopic and exposure images of balloons of different sizes at different angles in the AP, RAO/CAU, and RAO/CRA directions. In the AP direction, the differences in gray values of the background and the balloon images were above 24.0 in the exposure and stored fluoroscopic images of all balloon sizes with all devices. In the RAO/CAU direction, the differences in gray values of the background and the balloon images were above 21.9 in the exposure images of all balloon sizes with all devices. In the stored fluoroscopic images with device C, the differences in gray values of the background and the balloon images were 19.9 (balloon sizes ≤2.0 mm) and 14.5 (≤2.5 mm). With device B, the differences were 29.8 (balloon sizes ≤2.0 mm) and 32.6 (≤2.5 mm). With device A, the differences were 26.7 (balloon sizes ≤2.0 mm) and 24.6 (≤2.5 mm). In the RAO/CRA direction, the differences were above 22.0 in the exposure images of all balloon sizes with all devices. In the stored fluoroscopic images with device B, the differences were 17.8 (balloon sizes ≤ 2.0 mm) and 23.1 (≤2.5 mm).

## 4 DISCUSSION

### 4.A Dose settings of x‐ray diagnostic devices for circulatory organs

In the imaging condition with device A, the tube current was limited to 200 mA, and the tube current during fluoroscopy was 4.3 mA. The dose is determined by the mA value (tube current multiplied by irradiation time), and the manufacturer of device A may have set the low current value considering reducing the patient exposure. Further, the manufacturer may have tried to ensure the image quality by using digital filters as shown in Table 3, to be able to overcome the phenomenon with the tube voltage rises leading to blurring of images, which is a characteristic of the Flat Panel Detector (FPD). The characteristic of the dose settings with device B is to control the x‐ray dose with a large current, and the dose which patients are exposed to is controlled by eliminating soft rays using an additional filter. For device C, which uses a direct conversion style, FPD, it can be inferred that the dose is set to improve the sharpness by maintaining the tube voltage lower than for the other features involved, in the 70 kV range, and by taking advantage of the features of the direct conversion system.

**Table 3:** Types of digital and load filters by cardiovascular x‐ray diagnostic devices

Device	Fluoroscopy	Exposure	Additional filter
A	Harmonization	Harmonization	Without
	Rounding	Rounding	
	Edge enhance	Edge enhance	
	Brightness	Brightness	
B	FNR	FNR	With
	DRM	DRM	
C	Frequency filter	Frequency filter	With
	Recursive filter	Recursive filter	

### 4.B. Visual evaluation of balloons with different sizes at different imaging angles

In the visual evaluation, all the exposure images at the imaging angle AP scored 8 points with all devices. The dose rates of the exposure images showed about 1.3 times of difference between the lowest (device A) and the highest (device B) devices. Also, in device A, the visual evaluation scores with balloons with sizes other than 5.0 mm tended to decrease at the RAO/CAU and RAO/CRA angles. However, with devices B and C, even when the imaging angles were changed, the score showed 8 points like in AP. This is because there is a difference in the method of setting the image quality and the x‐ray output dose by the various facilities: device A performs visual evaluations at the AP angle which has the lowest load of object thickness, while devices B and C perform this at the angle which passes through the “thickest” part of the phantom (has the highest load of the object thickness) leading to the suggestion that the dose setting of the AP of devices B and C is unnecessarily high. Next, in the visual evaluations of stored fluoroscopic images, balloons with smaller diameters resulted in all devices receiving low evaluation scores. For the fluoroscopic dose rate, when assuming device A, which has the lowest dose setting, as the standard, the device B setting is 1.6 times and for device C it is about 2.2 times higher than that of device A. Usually, when the dose rate is high, the image quality improves, but the evaluation of the balloons with small diameters in devices B and C with the higher dose rate was lower than in device A. This is because device A controls the image quality by controlling the x‐ray dose. However, in devices B and C the signals of the balloons are flattened because these devices cut soft x‐rays using an additional filter, and this may lower the visual evaluation due to poor control of the image quality with the digital filter as shown in Table 3.

### 4.C Physical evaluation of balloons with different sizes at different imaging angles

In the physical evaluation of balloons with different sizes at different imaging angles, the AP was the highest score (gray value contrast) among all the balloon sizes, and the physical evaluation became poorer as the imaging angle became larger. This may be because the tube voltage increases and the image blurs due to the larger imaging angles than that of AP, eliminating the difference between the contrasts recorded with the contrast agent in the balloon and the gray value of the background. Further, it may also be because, as the balloon diameter decreases, the amount of contrast agent in the balloon decreases, and as a result the differences in the background gray value disappear when the contrast decreases.

**Figure 6:**
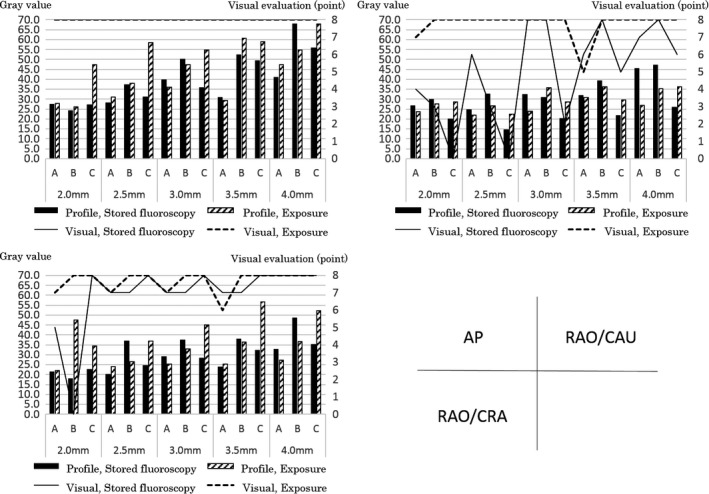
Graphs and plots of evaluations of both physical and visual evaluations at the same time points. Solid line: Visual, stored fluoroscopic image; Wavy line: Visual, exposure image; Left of *y*‐axis: gray value; Right of *y*‐axis: visual evaluation (point).

**Figure 7:**
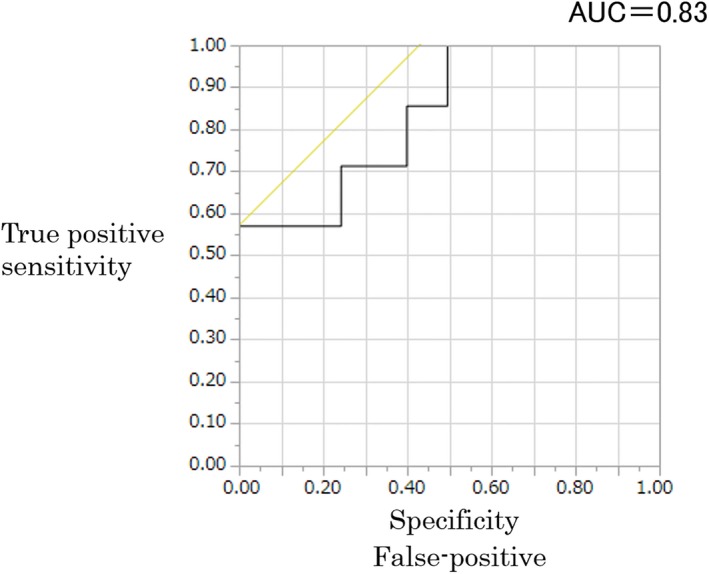
Results of receiver operating characteristic analysis obtained from the visual and physical evaluations by the Wilcoxon test.

**Figure 8:**
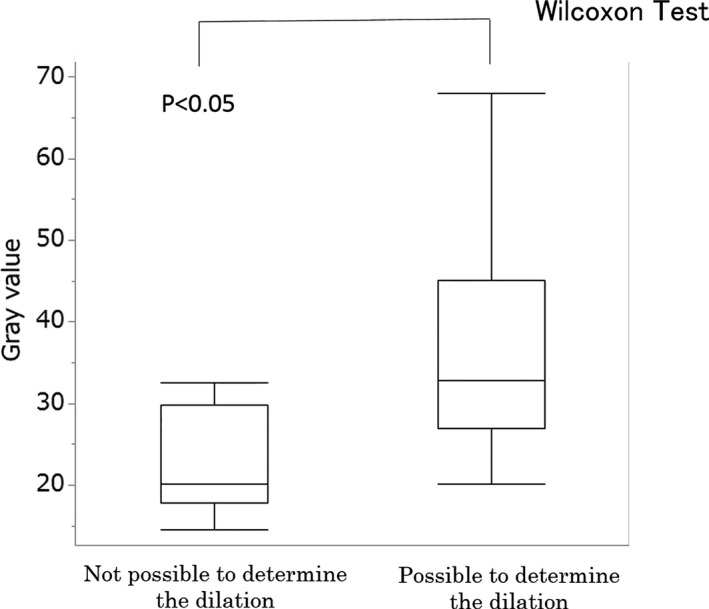
Results in gray values obtained from the visual and physical evaluations by the Wilcoxon test.

Figure 6 shows graphs obtained by evaluating both physical and visual evaluations plotted together. In the physical evaluation, when the gray value was below 20, lower values were also shown in the visual evaluations of the stored fluoroscopic images. For this reason, we performed a Receiver Operating Characteristic (ROC) analysis using the statistical analysis software Jamp 1.4. In the ROC analysis, the cutoff gray value of the balloons was 20.1, the value where the eight physicians who performed the visual evaluations suggested that they were unable to determine the balloon dilation. At this gray value, the Area Under the Curve (AUC) was 0.83, the sensitivity was 57.1%, the specificity was 100%, and the physical and visual evaluations agreed. Further, there was a significant difference in the gray value used for determining balloon dilation when the cutoff gray value of the balloon was set to 20.1 (Figs. 7 and 8).

## 5 CONCLUSIONS

This study finds that replacement of stored fluoroscopic images with exposure images requires 20.1 or higher gray value differences between the background and signal, using an acrylic phantom of 20 cm thickness (representing the abdomen of a human adult) a specific geometry. This suggests the conclusion that the gray value can be considered a useful index when using stored fluoroscopic images as exposure images.

## CONFLICT OF INTEREST

The authors declare no conflicts of interest associated with this manuscript.
